# Higher Rate of Tuberculosis in Second Generation Migrants Compared to Native Residents in a Metropolitan Setting in Western Europe

**DOI:** 10.1371/journal.pone.0119693

**Published:** 2015-06-10

**Authors:** Florian M. Marx, Lena Fiebig, Barbara Hauer, Bonita Brodhun, Gisela Glaser-Paschke, Klaus Magdorf, Walter Haas

**Affiliations:** 1 Department of Pediatric Pneumology and Immunology, Charité –Universitätsmedizin, Berlin, Germany; 2 Division of Global Health Equity, Brigham and Women’s Hospital and Harvard Medical School, Boston, United States of America; 3 Respiratory Infections Unit, Department for Infectious Disease Epidemiology, Robert Koch Institute, Berlin, Germany; 4 Centre for Tuberculosis, Public Health Office Berlin-Lichtenberg, Berlin, Germany; University of Otago, NEW ZEALAND

## Abstract

**Background:**

In Western Europe, migrants constitute an important risk group for tuberculosis, but little is known about successive generations of migrants. We aimed to characterize migration among tuberculosis cases in Berlin and to estimate annual rates of tuberculosis in two subsequent migrant generations. We hypothesized that second generation migrants born in Germany are at higher risk of tuberculosis compared to native (non-migrant) residents.

**Methods:**

A prospective cross-sectional study was conducted. All tuberculosis cases reported to health authorities in Berlin between 11/2010 and 10/2011 were eligible. Interviews were conducted using a structured questionnaire including demographic data, migration history of patients and their parents, and language use. Tuberculosis rates were estimated using 2011 census data.

**Results:**

Of 314 tuberculosis cases reported, 154 (49.0%) participated. Of these, 81 (52.6%) were first-, 14 (9.1%) were second generation migrants, and 59 (38.3%) were native residents. The tuberculosis rate per 100,000 individuals was 28.3 (95CI: 24.0–32.6) in first-, 10.2 (95%CI: 6.1–16.6) in second generation migrants, and 4.6 (95%CI: 3.7–5.6) in native residents. When combining information from the standard notification variables country of birth and citizenship, the sensitivity to detect second generation migration was 28.6%.

**Conclusions:**

There is a higher rate of tuberculosis among second generation migrants compared to native residents in Berlin. This may be explained by presumably frequent contact and transmission within migrant populations. Second generation migration is insufficiently captured by the surveillance variables country of birth and citizenship. Surveillance systems in Western Europe should allow for quantifying the tuberculosis burden in this important risk group.

## Introduction

In Europe and globally, tuberculosis remains an important public health challenge. An increasing share of the tuberculosis burden is associated with international migration [1, 2]. Currently, around one fourth of tuberculosis patients reported in the European Union/European Economic Area (EU/EEA) are of foreign origin [3]. In 2010, individuals of foreign country of birth and/or foreign citizenship accounted for the majority of tuberculosis patients in 8 of the 27 EU member states, reaching 70% in the United Kingdom, 71% in the Netherlands, and 89% in Sweden, for example [3].

Immigration to and within the EU is considered one of the major determinants for changing tuberculosis trends in Western European countries: In the United Kingdom, the increase in tuberculosis incidence observed between 1996 and 2005 was, at least partially, due to cases among foreign-born individuals, many of whom originate from countries with a high burden of the disease [4]. Progress toward elimination of tuberculosis in the Netherlands seems to be slowed down by an increasing proportion of foreign-born tuberculosis cases [5, 6] who are estimated to represent 85% of all incident cases in 2030 [6]. High rates of active tuberculosis in foreign-born children and adolescents have been observed in Stockholm [7].

Although migrants account for a considerable proportion of tuberculosis cases in Western Europe, studies have shown that transmission of *Mycobacterium tuberculosis* occurs mainly within migrant communities but rarely between migrants and the native population [8–10].

Despite the significance of migration for tuberculosis control in Europe [11], current knowledge is based primarily on routinely collected data. In line with current standards [3], surveillance systems across Europe commonly rely on variables for geographic origin, i.e. country of birth and citizenship, to quantify migration in tuberculosis cases; very few surveillance systems directly capture migration. These proxy variables for migration are subject to important limitations. Specifically, they fail to capture successive generations of migrants given that these are usually born in their country of destination and may as well hold a citizenship of that country. To our knowledge, prospective studies on the risk of tuberculosis in successive migrant generations in Europe are lacking.

In Western Europe, much of the tuberculosis burden accumulates in major cities [12, 13] where effective tuberculosis control requires a closer focus on migrants, including undocumented migrants [14], and other vulnerable groups [15–17]. We conducted this study in Berlin, the German capital city, with 3.4 million residents, 25% of whom are estimated to be migrants [18], and with tuberculosis rates that are constantly above national average (9.3 vs. 5.3 cases per 100,000 population in 2011, respectively) [19]. The purpose of this study was to investigate migration patterns among tuberculosis cases notified in Berlin, and to compare tuberculosis rates of second generation migrants (and their parent generation) to that in the native resident population. We further aimed to investigate the sensitivity of the current notification system to capture the status of migration among notified tuberculosis cases.

## Methods

### Study population

Individuals eligible for this study were notified as cases of tuberculosis to the Berlin health authorities between 1^st^ November 2010 and 31^st^ October 2011. The definition of a tuberculosis case in Germany is based on the decision made by the attending physician to start a full course of anti-tuberculosis treatment [19]. Notification is mandatory for all tuberculosis cases who are categorized as either clinically confirmed, clinically confirmed with an epidemiological link to another confirmed case, or laboratory confirmed [19].

Eligible individuals were selected from the electronic notification system which is operated jointly by public health authorities at community and federal level, and included if they gave informed consent to be interviewed. Participants included tuberculosis patients diagnosed in the penitentiary system. Individuals were not considered for the study if they were initially notified elsewhere (i.e. transferred in), if their diagnosis was established post-mortem, or if the case report was withdrawn due to unfounded diagnosis within three months of reporting.

### Study definitions

The study population was divided into three study groups: (i) First generation migrants were all individuals who were foreign-born and immigrated to Germany after the year 1949 (before 3^rd^ October 1990: immigration either to the Federal Republic of Germany or to the German Democratic Republic). (ii) Second generation migrants were born in Germany to at least one parent being a first generation migrant or of foreign citizenship. These definitions were in accordance with those used for the German microcensus 2011 (Federal Statistical Office) [18]. (iii) Native residents included all individuals who did not meet the above definitions. The term ‘foreign origin’ in this study denotes individuals with either a foreign country of birth or a foreign citizenship.

### Study design and data collection

A prospective cross-sectional study was conducted. Eligible individuals were informed about the study by the attending social worker of the Berlin Centre of Tuberculosis either face-to-face or via a letter. Letters were produced in German, English, French, Turkish, Russian and Arabic. If consent was given to be contacted for the study, contact details were transferred to the study team. Following informed verbal consent (see: ethics statement), face-to-face interviews were conducted by a trained study nurse, using a standardized questionnaire. The questionnaire included demographic information (such as date and place of birth), information about migration history of patients and their parents, and maternal and household language use. Patients with insufficient German language skills were interviewed with assistance of certified interpreters.

### Data sources and management

Data obtained via the interviews were entered into a study database using double-entry verification. The study database was linked at the Robert Koch Institute with the standard notification data for Berlin of the same period, using a list of unique identifiers administered by the Centre for Tuberculosis. Population-based data on the number of first and second generation migrants and native residents in Berlin were obtained from the German microcensus 2011 [18].

### Data Analysis

STATA 10.1 statistical application (Stata Corp, College Station, TX, USA) was used for data analysis. The response rate for the study was determined by comparing the number of patients enrolled to the number of tuberculosis cases notified in the study period. A non-response analysis was conducted on the basis of notification data, in order to assess the effect of participation on tuberculosis rates in each of the three study groups. Variables were included in a multivariable logistic regression model according to the strength of their association, using a step-wise forward technique. Likelihood ratio test statistics were calculated to identify variables independently associated with study participation at significance level α = 0.05, and to assess potential interaction.

Proportions of first-, second generation migrants and native residents and their 95% confidence intervals (CI) in the study sample were calculated. Tuberculosis rates and rate ratios for each of the study groups were calculated as follows: The proportions of first- and second generation migrants and native residents observed in the study sample were applied to the total number of tuberculosis cases notified, to calculate the expected numbers of tuberculosis patients for each of the study groups. Rate denominators for the three study groups were derived from the microcensus. Rate-ratios were calculated for first- and second generation migrants, using the tuberculosis rate among native residents as the baseline.

In order to assess for non-response bias, tuberculosis rates were re-calculated on the basis of group proportions weighted for study participation using inverse probability weights [20] derived from the logistic regression model on study participation (see above).

To investigate how complete first and second generation migration was captured in the German notification system we calculated sensitivity estimates for the notification data variables country of birth and citizenship using our study results as a reference. “Sensitivity” was defined as the number of individuals considered as migrants when a particular variable was used divided by the true number of migrants (first and second generation) ascertained through the interviews. For example, the sensitivity to detect migration by the variables country of birth and citizenship combined was calculated as the number of study participants with either foreign country of birth or foreign citizenship (according to the notification data) divided by the total number of first and second generation migrants identified through the study.

### Ethics statement

The study was approved by the Ethics Committee of the Charité –Universitätsmedizin Berlin (№ EA2/126/10). A detailed procedure of information and verbal informed consent that takes into account the specific needs of eligible patients in our setting was developed. The patients’ age and their capacity and ability to understand the purpose and essential information of the study were assessed by the attending social worker (upon pre-information) and again by the interviewer (and interpreter). A standardized information and consent protocol was used to document the patient’s comprehension of the study and verbal consent. In individuals with reduced capacity or ability to provide informed verbal consent and in children under 18 years of age, we sought to obtain informed consent from and to conduct the interview with a parent or legal guardian. Additionally, assent to participate was sought in children aged between 10 and 17 years. We decided to obtain verbal instead of written consent for the following reasons: We considered that immigrants may feel uncomfortable to provide written informed consent, in particular if they were staying in Germany without legal residence permit. Further, verbal informed consent allowed us to offer eligible patients to be informed (and interviewed) via phone in case they were unable to come to the study centre or uncomfortable to receive visitors. The procedure of verbal informed consent including the consent protocol was approved by the Ethics Committee.

Access and linkage of notification data with the research database was approved by the data protection officers of the Charité –Universitätsmedizin Berlin and the Robert Koch Institute.

## Results

### Characteristics of study participants

**Fig 1:**
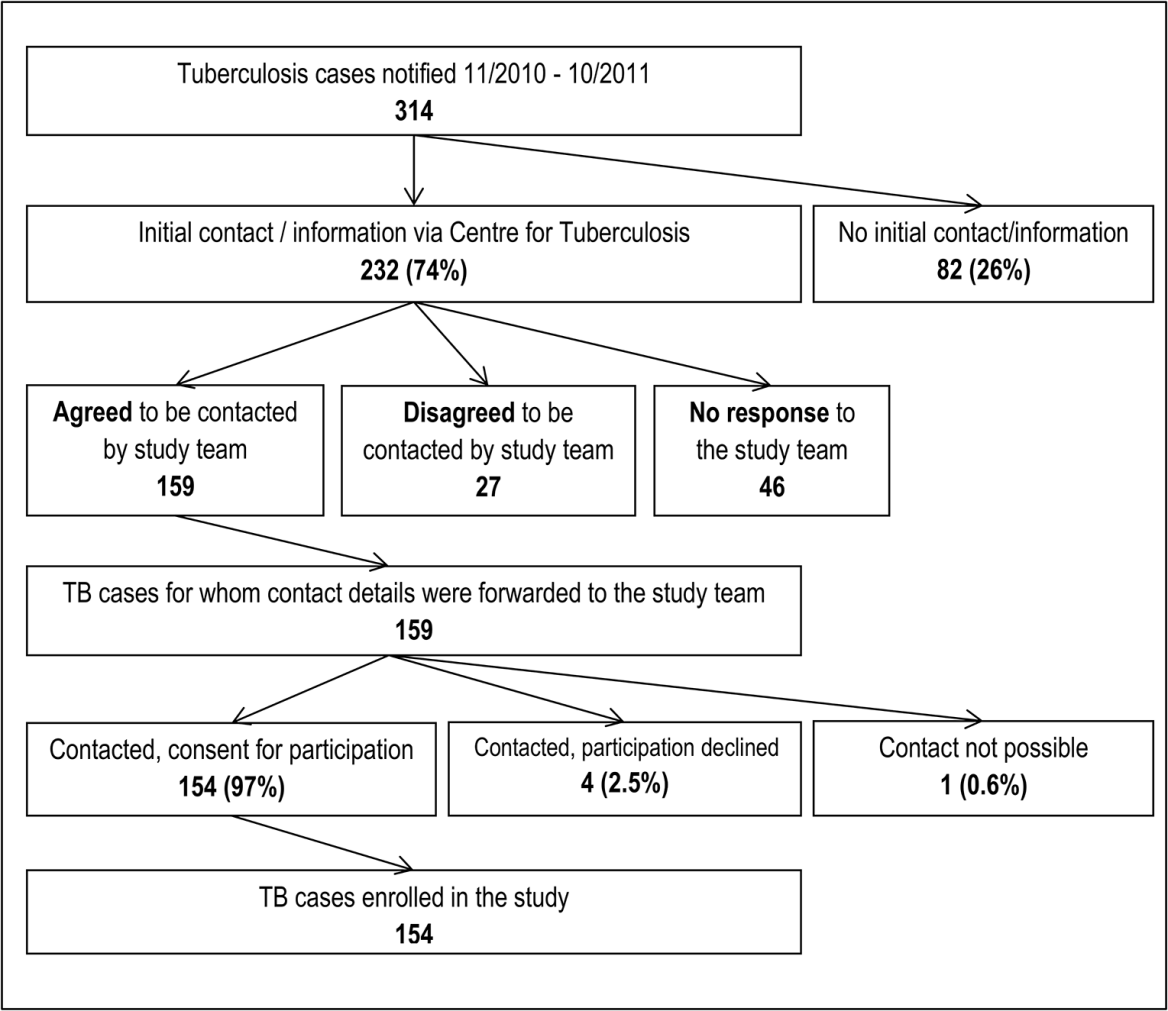
Overview of the study recruitment.

A total of 314 tuberculosis cases, 188 (59.9%) male, at a median age of 45 years (inter-quartile range [IQR]: 32–62 years), were reported in Berlin between November 2010 and October 2011, equivalent to 9.0 cases per 100,000 population. Of these, 154 (49.0%) were enrolled in the study (Fig 1). Reasons not to enrol eligible participants included failure to contact patients who either moved or died or were in bad general condition, or for reasons not documented by the social workers; further, patients being contacted via letter but not responding, and patients not consenting to participate (Fig 1). Age equal to or greater than 65 years was independently associated with lower study participation (Tables 1 and 2). Further, patients of foreign origin were less likely to participate if they were cases of extrapulmonary tuberculosis (likelihood-ratio test for interaction: P = 0.02; Table 2). The main characteristics of the study participants are shown in Table 3.

**Table 1:** Univariable regression analysis of study participation (N = 314 tuberculosis cases notified in Berlin, 2010–2011).

Variable	Variable category	Cases notified	Cases participated	Univariable analysis	
		*N (%)*	*N (%)*	*OR (95% CI)*	P-value
**Sex**					0.18
	Female	126 (100)	56 (44.4)	1	
	Male	188 (100)	98 (52.1)	1.36 (0.87–2.14)	
**Age**					0.02
	0–24	43 (100)	20 (46.5)	0.70 (0.35–1.42)	
	25–44	112 (100)	62 (55.4)	1	
	45–64	88 (100)	48 (54.6)	0.97 (0.55–1.70)	
	65+	71 (100)	24 (33.8)	0.41 (0.22–0.76)	
**Site of disease** ^1^					0.003
	Pulmonary	231 (100)	125 (54.1)	1	
	Extrapulm.	83 (100)	29 (34.9)	0.46 (0.27–0.77)	
**Bacteriological confirmation**					0.37
	No	93 (100)	42 (45.2)	1	
	Yes	221 (100)	112 (50.7)	1.25 (0.77–2.03)	
**Country of birth**					0.09
	Germany	137 (100)	75 (54.7)	1	
	Other	173 (100)	78 (45.1)	0.68 (0.43–1.06)	
**Citizenship**					0.14
	German	149 (100)	80 (53.7)	1	
	Other	161 (100)	73 (45.3)	0.72 (0.46–1.12)	
**Origin** ^2^					0.12
	Non-foreign	134 (100)	73 (54.5)	1	
	Foreign	176 (100)	80 (45.5)	0.70 (0.44–1.09)	

OR = Odds ratio; CI = Confidence interval; Extrapulm. = Extrapulmonary

^1^ Concurrent pulmonary and extrapulmonary disease manifestations were classified as pulmonary.

^2^ Origin is a combined indicator of (foreign) country of birth and/or (foreign) citizenship.

**Table 2:** Multivariable regression analysis of study participation (N = 310 tuberculosis cases^1^ notified in Berlin, 2010–2011).

Variable	Variable category	Cases notified	Cases participated	Multivariable analysis	
		*N (%)*	*N (%)*	*OR (95% CI)*	P-value
**Age**					<0.001
	0–24	43 (100)	20 (46.5)	0.59 (0.28–1.23)	
	25–44	112 (100)	62 (55.4)	1	
	45–64	88 (100)	48 (54.6)	0.76 (0.41–1.39)	
	65+	71 (100)	24 (33.8)	0.25 (0.12–0.51)	
**Site of disease and origin** ^2^					0.02^4^
	Pulmonary, Non-foreign	101 (100)	57 (56.4)	1	
	Pulmonary, Foreign	128 (100)	68 (53.1)	0.64 (0.36–1.13)^3^	
	Extrapulm., Non-foreign	33 (100)	16 (48.5)	0.99 (0.43–2.28)^3^	
	Extrapulm., Foreign	48 (100)	12 (25.0)	0.18 (0.06–0.51)^3^	

The model takes into account interaction between site of disease and foreign origin. OR = Odds ratio; CI = Confidence interval; Extrapulm. = Extrapulmonary.

^1^Foreign origin not determined for N = 4 notified cases (no data for country of origin and citizenship)

^2^Interaction between site of disease and origin; origin is a combined indicator of (foreign) country of birth and/or (foreign) citizenship.

^3^Stratum-specific odds ratios are shown for site of disease and origin

^4^P-value for site of disease and origin represents test for interaction between site of disease and origin

**Table 3:** Main characteristics of the study participants (N = 154 tuberculosis patients).

Variable	Variable category	ALL	1st generation migrants	2nd generation migrants	Native residents
**Total**		154	81	14	59
**Median age (years)** ^1^	-	43 (31–55)	40 (29–50)	27.5 (9–36)	52 (42–68)
**Sex**	Male	99 (64%)	54 (67%)	8 (54%)	37 (63%)
**Site of disease**	Pulmonary	125 (81%)	68 (84%)	9 (64%)	48 (81%)
**Bacteriology**	Confirmed	112 (73%)	57 (70%)	10 (71%)	45 (76%)
**Country of birth**	Germany	71 (46%)	0 (0.0%)	14 (100%)	57 (97%)^2^
**Citizenship**	German	81 (53%)	11 (14%)	11 (79%)	59 (100%)

^1^Median age: data in brackets show inter-quartile range.

^2^Only 57 of the 59 native residents were born in Germany. Two patients were born in a foreign country but immigrated before 1950 to Germany and, according to the study definitions, were allocated to the group of native residents.

### First and second generation migrants

Of the 154 tuberculosis patients enrolled in the study, 81 (52.6%; 95%CI: 44.6%–60.4%) were first generation migrants according to the study definition, 14 (9.1%; 95%CI: 5.4%–14.8%) were second generation migrants, and the remaining 59 (38.3%; 95%CI: 30.9%–46.3%) patients were native residents.

**Fig 2:**
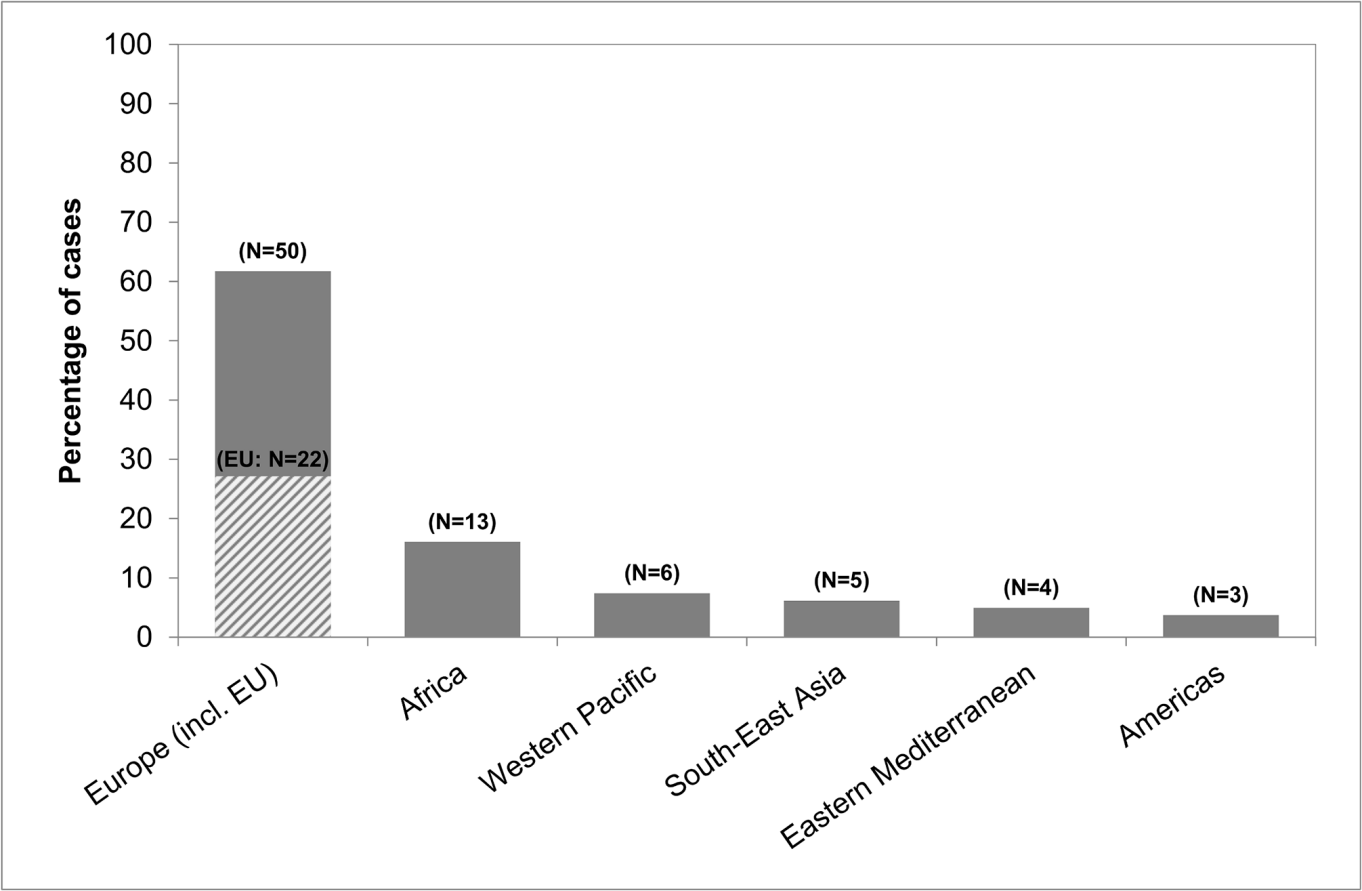
Origin of first generation migrants enrolled in the study by WHO Region (N = 81). Numbers in brackets show total numbers of cases. EU = European Union: shaded upward diagnoal.

**Fig 3:**
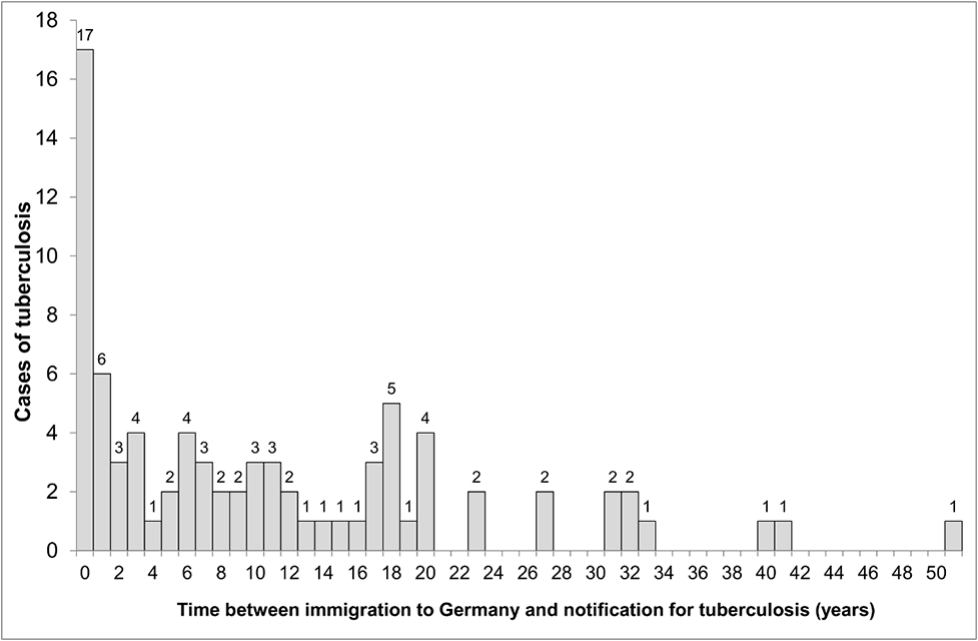
Distribution of first generation migrants by time since their immigration to Germany (N = 81).

First generation migrants were younger than native residents (P<0.001), and two-third were male (Table 3). They had emigrated from Russia (10 cases; 12.3% of all patients), Turkey (7; 8.6%), Bulgaria (6; 7.4%), Cameroon (5; 6.2%), Poland (5; 6.2%), and 31 other countries (48; 59.3%). Immigrant patients by region of origin are shown in Fig 2. The majority of first generation migrants, 47 of 81 (58.0%), had immigrated less than 10 years before being notified (28.4% less than two years), and 69 (85.2%) had immigrated less than 20 years before being notified (Fig 3). The median time between immigration and notification was 8 years (IQR: 1–18 years). Seventy-eight provided information about the personal reason for immigration: 37 (47.4%) had joined family already living in Germany, 13 (16.7%) were asylum seekers, 12 (15.4%) had immigrated as guest workers, 9 (11.5%) were guest or exchange students, 6 (7.7%) had a status of late repatriates, and one (1.3%) was au-pair. Of 80 first generation migrants providing information, twenty-three (28.8%) reported to visit their home country at least once a year, 26 (32.5%) less than once a year, and 31 (38.8%) had not been visiting their home country since immigration.

Second generation migrants were younger than first generation migrants (P = 0.046) and native residents (P = 0.002), and 8 of 14 (57.1%) were male (Table 3). Eleven of 14 had a German citizenship. All 14 had at least one parent who had immigrated to Germany. Countries of origin belonged to the WHO Region of Europe (10 of 14; EU: 8), South-East Asia (2), Africa (1) and the Americas (1). Eight of 14 second generation migrants reported German to be their mother tongue and to speak exclusively German in the household.

### Tuberculosis rates and rate ratios

**Fig 4:**
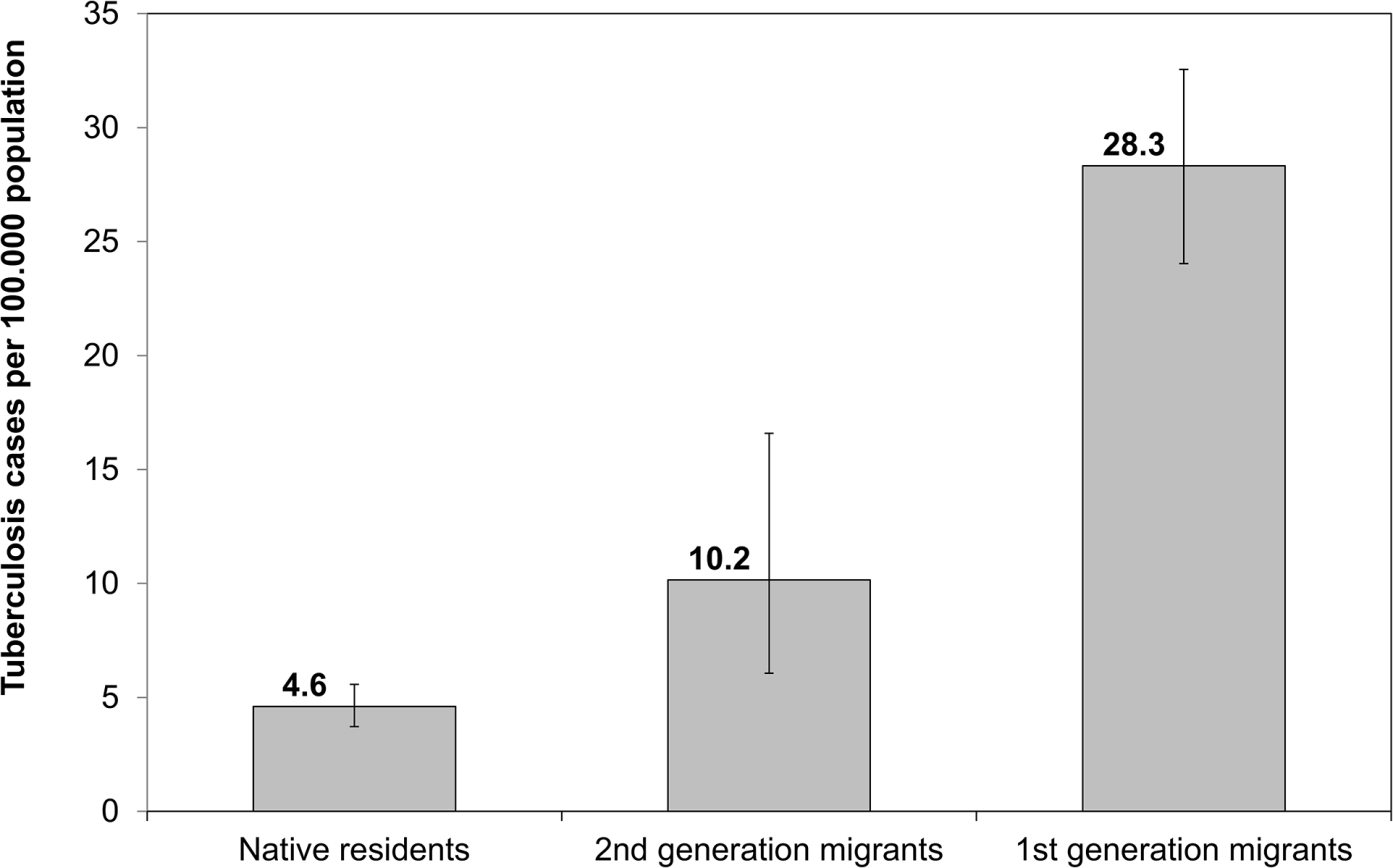
Comparison of tuberculosis rates among first and second generation migrants, and native residents. Error bars show 95% confidence intervals.

The crude tuberculosis rate per 100,000 individuals was 28.3 (95CI: 24.0–32.6) in first-, 10.2 (95%CI: 6.1–16.6) in second generation migrants, and 4.6 (95%CI: 3.7–5.6) in native residents (Fig 4). The crude rate ratios for second and first generations migrants were 2.2 (95% CI: 1.3–3.6) and 6.1 (95% CI: 5.2–7.1).

### Assessment for non-response bias

Study participation had little effect on the tuberculosis rate estimates: The tuberculosis rates weighted for study participation were 31.0 (95CI: 27.7–34.3) in first-, 9.2 (95%CI: 6.1–13.7) in second generation migrants, and 4.1 (95%CI: 3.4–4.8) in native residents (each per 100,000 individuals). The weighted rate ratios for second and first generations migrants were 2.2 (95%CI: 1.5–3.3) and 7.6 (95%CI: 6.7–8.3).

### Sensitivity to assess the status of migration via notification data

Notification data for country of birth and citizenship were available for 153 of the 154 tuberculosis patients enrolled in the study. Table 4 shows a cross-comparison of notification data for the two variables with either migration (first or second generation) or native resident as ascertained by the study.

**Table 4:** Cross-comparison of country of birth/citizenship (according to notification data) vs. status of migration ascertained through the study (N = 153 patients)^1^.

Variable	*Variable Category*	*Migrants* ^*2*^	*Native residents*	*TOTAL*
**Country of Birth**				
	Germany	16 (17.0%)	59 (100.0%)	75 (49.0%)
	Foreign	78 (83.0%)	0 (0.0%)	78 (51.0%)
	TOTAL	94 (100.0%)	59 (100.0%)	153 (100.0%)
**Citizenship**				
	German	21 (22.3%)	59 (100.0%)	80 (52.3%)
	Foreign	73 (77.7%)	0 (0.0%)	73 (47.7%)
	TOTAL	94 (100.0%)	59 (100.0%)	153 (100.0%)

^1^Country of birth and citizenship ascertained via the routine notification system may differ from country of birth and citizenship ascertained through the study. Notification data for country of birth and citizenship were unavailable for N = 1 study participant.

^2^Migrants include the first and second generation according to the study definitions.

The sensitivity to detect any status of migration (first or second generation) was 83.0% (95%CI: 73.9%–89.4%) when using the variables country of birth, 77.7% (95%CI: 68.0%–85.0%) when using citizenship, and 85.1% (95%CI: 76.3%–91.0%) when using both variables combined (foreign origin).

Using both variables combined (i.e. either foreign country of birth or foreign citizenship) resulted in 95.0% (95%CI: 87.3%–98.1%) sensitivity to detect first- and 28.6% (95%CI: 10.6%–57.4%) to detect second generation migration.

## Discussion

This study shows that in Berlin, one of the European capital cities, second generation migrants have a 2-fold higher rate of tuberculosis compared to native (non-migrant) residents. The tuberculosis rate appears to be gradually higher from native residents to second to first generation migrants. Both first and second generation migrants together account for almost two-thirds of tuberculosis cases, a finding that is in line with trends in other major European cities, underscoring that migrants (and their families) constitute one of the most important risk groups for tuberculosis control in Western Europe nowadays [4].

To date, very few studies in Europe and elsewhere have investigated tuberculosis in successive generations of migrants. In a series of prospective studies of childhood tuberculosis in Sweden more than 30 years ago, Romanus showed that tuberculosis was about eight to ten times more common in non-BCG-immunized children born in Sweden to foreign parents than in children born to Swedish parents [21, 22].

Our prospective study suggests that second generation migrants are at higher risk of tuberculosis compared to native residents: Several factors may explain this finding: Second generation migrants may be at increased risk of infection compared to native residents due to more frequent contact to/mixing with individuals at higher risk of active tuberculosis, such as the parent generation of migrants. Crowded conditions in asylum or migrant shelters [23, 24] may accelerate this risk. Further, visiting their family’s country of origin [25, 26] may contribute to a higher risk of tuberculosis in second (and first) generation migrants compared to native residents, particularly in those visiting countries with a high prevalence of the disease. Upon infection, second generation migrants might incur an increased risk of disease progression compared to native residents due to socio-economic, lifestyle and nutritional factors.

The high risk of tuberculosis in individuals with a personal history of immigration to Western Europe, such as asylum seekers, is well documented [27–29]. We found that most first generation migrants with tuberculosis immigrated to Germany in more recent years, a finding that is consistent with previous studies that have documented a continuous decline but persistently high risk of tuberculosis over time since immigration [1, 30–32].

We show that under the prevailing German notification system second generation migrants are not sufficiently captured. The system includes data for country of birth and citizenship, which, as we show, allow for capturing first generation migrants but are insufficient to reasonably distinguish second generation migrants from the resident population, rendering tuberculosis monitoring and evaluation in this particular group impossible.

Our study was conducted in the complex environment of city-wide tuberculosis health care services and has several limitations. We were unable to include more than half of eligible patients in this study. Data protection and confidentiality issues required a rigorous recruitment strategy, where patients could only be contacted once within a pre-specified time window and informed about the study by the attending social worker. Non-response was due mainly to failure to contact (or receive feedback) from eligible tuberculosis patients. We tried to avoid non-response bias by ensuring the voluntary nature and confidentiality of the interviews, and by providing multilingual information and interpretation. Weighting for factors associated with lower participation resulted in only minor changes to notification rates estimated in our study, suggesting that non-response bias is unlikely to explain the observed results. However, absolute rates and rate-ratios have to be interpreted with caution because the possibility of non-response bias cannot be fully excluded. Our study aimed to investigate the frequency of tuberculosis in subpopulations, but it does not allow for estimating risk at the individual level given that individuals may be heterogeneous in terms of other risk factors for tuberculosis, e.g. socio-economic determinants, risk behaviour such as smoking and alcohol or drug abuse, or HIV co-infection [33]. Further, sample size did not allow us to stratify the rate of tuberculosis in immigrants by different countries or areas of origin. Moreover, we were unable to consider incident tuberculosis cases not known to the health care system. Hence, rates estimated in this study are based on the number of notified cases and therefore represent underestimates of true incidence.

## Conclusions

In this large metropolitan setting in Western Europe, second generation migrants are at higher risk of tuberculosis compared to native (non-migrants) residents. Their elevated risk is likely related to the much higher risk in first generation migrants. Given the long period between immigration and disease notification in the latter (median: 8 years), it is unlikely that at-entry screenings for tuberculosis alone are currently effective. Instead, making primary health care accessible and affordable for migrant families, particularly for refugees and asylum-seekers, could have a greater impact on tuberculosis control [34]. Strengthening of primary health care is needed to increase provider’s awareness of symptoms and risk factors for tuberculosis and to provide information and care that is culturally and socially sensitive. A more comprehensive approach for tuberculosis prevention and management for first generation migrants at the primary care level will likely benefit tuberculosis control in successive migrant generations. We speculate that care providers should additionally consider screening for latent infection in second generation migrants.

We show that migration as an important determinant for tuberculosis in Western Europe is incompletely captured by the surveillance variables country of birth and citizenship because these variables largely fail to capture second generation migration. Surveillance systems in Western European countries should allow for quantifying the tuberculosis burden in this important risk group. By the time this study was conducted, Germany adapted routine recording and reporting to include information about parental migration history of tuberculosis cases.
